# Serostatus testing and dengue vaccine cost–benefit thresholds

**DOI:** 10.1098/rsif.2019.0234

**Published:** 2019-08-21

**Authors:** Carl A. B. Pearson, Kaja M. Abbas, Samuel Clifford, Stefan Flasche, Thomas J. Hladish

**Affiliations:** 1 Department of Infectious Disease Epidemiology, London School of Hygiene and Tropical Medicine, London, UK; 2 Centre for the Mathematical Modelling of Infectious Diseases, London School of Hygiene and Tropical Medicine, London, UK; 3 South African Centre for Epidemiological Modelling and Analysis, Stellenbosch University, Stellenbosch, South Africa; 4 Department of Biology and Emerging Pathogens Institute, University of Florida, Gainesville, FL, USA

**Keywords:** dengue, CYD-TDV, cost–benefit analysis, seroprevalence, Dengvaxia

## Abstract

The World Health Organization (WHO) currently recommends pre-screening for past infection prior to administration of the only licensed dengue vaccine, CYD-TDV. Using a threshold modelling analysis, we identify settings where this guidance prohibits positive net-benefits, and are thus unfavourable. Generally, however, our model shows test-then-vaccinate strategies can improve CYD-TDV economic viability: effective testing reduces unnecessary vaccination costs while increasing health benefits. With sufficiently low testing cost, those trends outweigh additional screening costs, expanding the range of settings with positive net-benefits. This work highlights two aspects for further analysis of test-then-vaccinate strategies. We found that starting routine testing at younger ages could increase benefits; if real tests are shown to sufficiently address safety concerns, the manufacturer, regulators and WHO should revisit guidance restricting use to 9-years-and-older recipients. We also found that repeat testing could improve return-on-investment (ROI), despite increasing intervention costs. Thus, more detailed analyses should address questions on repeat testing and testing periodicity, in addition to real test sensitivity and specificity. Our results follow from a mathematical model relating ROI to epidemiology, intervention strategy, and costs for testing, vaccination and dengue infections. We applied this model to a range of strategies, costs and epidemiological settings pertinent to CYD-TDV. However, general trends may not apply locally, so we provide our model and analyses as an R package available via CRAN, denvax. To apply to their setting, decision-makers need only local estimates of age-specific seroprevalence and costs for secondary infections.

## 1. Introduction

Global incidence of symptomatic dengue is estimated at 50–100 million cases annually, with burden concentrated in low- and middle-income countries [1,2]. While many organizations advocate various dengue control measures, these settings have limited resources and many competing options to improve quality of life; thus, potential control efforts must be prioritized. One option, the only currently licensed dengue vaccine, CYD-TDV (commercially: Dengvaxia), presents a complicated assessment: there is potential benefit, but also safety risks and associated mitigation costs.

Dengue infections elicit complex immune responses, particularly in regions where people typically experience multiple infections. There are four known dengue serotypes; infection by one confers apparently lifelong immunity to it, and temporary immunity to others. Disease threat varies substantially by infection number: primary infections are generally asymptomatic, and when symptomatic are rarely severe; secondary infections are more often symptomatic, and more often severe; and post-secondary infections are almost always asymptomatic. This pathogenicity pattern is caused by antibody-dependent enhancement [3,4]. To avoid enhancement, vaccine development has focused on products effective against all serotypes. Though CYD-TDV initially appeared to achieve this goal [5,6], subsequent work concluded that the vaccine acted more like a silent natural dengue infection [7–11]: enhancing disease risk in seronegative (i.e. no prior dengue infection) recipients while being efficacious for previously infected recipients [12]. These disparate outcomes pose ethical challenges.

A multi-model comparison study estimated that using CYD-TDV in high-burden settings would reduce both moderate and severe cases overall [13]. These findings informed initial recommendations by the World Health Organization (WHO) to consider CYD-TDV for settings with high seroprevalence in the target age for routine vaccination, with a minimum target age of 9 years old to increase the likelihood of past infection [14].

Continuing observation in trial populations confirmed increased risk of severe outcomes in seronegative recipients [15]. The Strategic Advisory Group of Experts on Immunization (SAGE) suggested avoiding this risk by verifying prior infection with serological testing [16] and WHO revised its recommendations accordingly [17,18]. Practically, the revised guidance necessitates a point-of-care rapid diagnostic test (RDT); as of July 2019, no such test exists, precluding test-then-vaccinate strategies.

At US$78 per vaccinated individual, CYD-TDV is a marginal investment in many settings [13], so adding costs would seemingly decrease its attractiveness. But screening can plausibly optimize use, limiting vaccination to the individuals likely to benefit. To provide a decision tool for such investment, we developed a model of the relationship between three pertinent costs: secondary infections, vaccination and testing. Local decision-makers can use this approach for their specific circumstances to determine if CYD-TDV is worth further consideration. The model has deliberately generous assumptions, providing a simple way to reject CYD-TDV for a region, but additional work, using more realistic assumptions, is required to determine whether CYD-TDV is sufficiently beneficial.

Using this model, we found that test-then-vaccinate strategies generally provide health benefits compared with both non-vaccination and vaccination without testing, but not necessarily outweighing the additional costs. We evaluate the balance of benefits and costs using return-on-investment (hereafter ROI; net benefit per unit cost), which also enables comparison against other development options.

We found that test-then-vaccinate strategies typically yield their highest ROI when testing starts younger than the currently recommended 9 years. We also found ROI improves with periodic re-testing of initially seronegative individuals.

## 2. Methods

We derive ROIs using two limiting assumptions. We ignore transmission, and thus indirect vaccination benefits, and assume interventions work deterministically. Treating dengue incidence like an environmental risk is justified in endemic settings, given the predicted limited impact on transmission from CYD-TDV [13]. Assuming that the intervention is deterministic provides upper benefit limits. Because more realistic assumptions will reduce benefits, local authorities may reject CYD-TDV (e.g. for insufficient ROI) with this framework, but would need a more detailed model (e.g. incorporating test sensitivity and specificity based on real trials, incomplete vaccine efficacy in seropositive recipients) to justify positive ROI estimates.

The electronic supplementary material provides derivations; the R package, denvax, implements the analyses [19].

### 2.1. Dengue disease and CYD-TDV models

**Figure 1.**
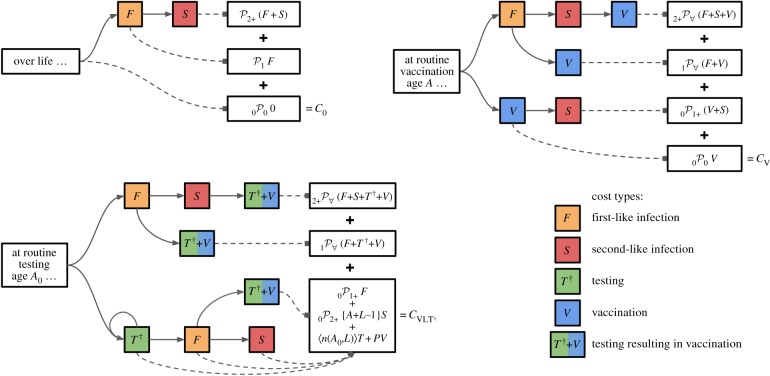
Lifetime outcomes by intervention. Trajectories shown for no vaccination, vaccination without testing and vaccination with multiple testing. Each path represents a possible life history, resulting in health outcome and intervention costs weighted by share of population following that path. For detailed branching probabilities, see electronic supplementary material, figures S2–S4 and S7–S8. (Online version in colour.)

We represent dengue disease with three infection outcomes—primary like, secondary like and post-secondary like—each with cost reflecting their respective disease risk and severity. Note that these average costs include all outcomes, from asymptomatic infections to death. We assume secondary infections have the highest cost, and post-secondary infections have zero cost. We assume CYD-TDV acts like a silent natural infection, preventing one of these outcomes, and testing reveals an individual’s infection history. Figure 1 shows life trajectories under different interventions.

### 2.2. Vaccination, testing and cost model

We calculate individual lifetime costs, using average primary and secondary infection costs (*F*, *S*) weighted by relevant life history probabilities denoted ${\;}_{N}P_{X}\left\{A\right\}$ (probability of *N* out of *X* lifetime infections by testing age *A*). Costs also include testing and, potentially, vaccination (*T*, *V*) during the intervention window. Strategies are defined by initial testing age and maximum allowed tests (*L*) at a rate of one per year. The model denotes the seroconversion probability between age *A* and *A* + 1 as C{A}, which determines the average test count. We estimate these probabilities with the exposure model described in the next section. We considered two test mechanisms: *binary*, detecting only the presence of past infection, and *ordinal*, detecting the number of past infections. Because the ordinal test also eliminates vaccination costs for people with multiple past infections, it is always more effective (ignoring cost). Though ordinal tests are theoretically better, we expect binary tests are more realistic, and assumed them for our general results.

Using these assumptions, we derived equation (2.1). The left side has intervention costs: weighted average number of tests administered, 〈*n*(*A*, *L*)〉 (equation (2.2)), and probability of vaccination, $P_{V}^{†}\{A+L-1\}$ (equation (2.3)), for a particular strategy. The right side is the lifetime difference in health costs. For all interventions with testing, first infection cost, *F*, cancels. Therefore, we can generalize across settings by expressing intervention costs relative to secondary infection costs: *ν* = *V*/*S* and *τ* = *T*/*S*.

2.1 $$\langle n(A,L)\rangle \tau^{†}-P_{V}^{†}\{A+L-1\}\nu \leq (P_{2^{+}}-{\;}_{0}P_{2^{+}}\{A+L-1\}+{\;}_{2^{+}}P_{\forall }),\;$$

2.2 $$\left\langle n(A,L)\rangle =1+{\;}_{0}P_{\forall }\{A+L-1\}(L-1)+\sum_{i=0}^{L-2}C{\left\{A+i\right\}}_{0}P_{1^{+}}\{A+i\}(i+1\right)$$

2.3 $$\mathrm{and}\;P_{V}^{†}\{A+L-1\}=1-{\;}_{0}P_{\forall }\{A+L-1\}.\;$$

ROI is the net benefits (difference in health outcome costs minus the intervention cost) per intervention cost,

2.4 $$\frac{P_{2^{+}}-{\;}_{2^{+}}P_{\forall }-{\;}_{0}P_{2}\{A+L-1\}}{\langle n(A,L)\rangle \tau^{†}-P_{V}^{†}\{A+L-1\}\nu }-1\geq 0,$$

for positive returns.

To identify circumstances where adding testing increases intervention benefits, we compared vaccination with and without testing, which produces a similar equation, but which depends on *F*,

2.5 $$\langle n(A,L)\rangle \tau^{*}+(1-P_{V^{*}}\{A+L-1\})\nu \leq ({\;}_{0}P_{1^{+}}-{\;}_{0}P_{2^{+}}\{A+L-1\})-{\;}_{0}P_{1^{+}}\frac{F}{S}.$$

### 2.3. Dengue infection and intervention model

We model dengue exposure in annual increments: each year, an individual is potentially exposed. We divide the population into risk groups, low and high. Thus, the exposure model has three parameters: *p*_*H*_, population fraction at high risk; and the annual avoidance probability for low (*s*_*L*_) and high (*s*_*H*_) risk groups. The probability of being seropositive at age *A* is thus

2.6 $$P_{+}\{A\}=\rho_{H}(1-s_{H}^{A})+(1-\rho_{H})(1-s_{L}^{A}).$$

We use a maximum likelihood approach to fit this model to age-seroprevalence data, then use the parameters to simulate exposure histories. For each life-year, we uniformly select one of the four serotypes to cause exposures, then probabilistically expose individuals based on their risk. Exposure leads to infection, unless the individual was (i) previously infected by this serotype or (ii) infected in the previous year. We estimate lifetime outcome probabilities by aggregating many simulated individual histories.

## 3. Results

In these sections, we demonstrate the types of analyses available to the decision-makers using the models described above. Because each region will differ, in costs, epidemiology and decision criteria, we cannot present a singular recommendation. We illustrate detailed application of the framework with two examples including particular epidemiology and costs, but these are demonstrations, not conclusive results.

### 3.1. General trends

**Figure 2.**
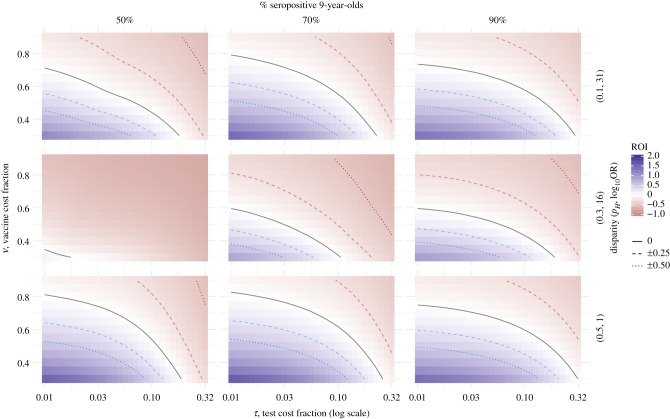
Lifetime ROI surfaces across settings. Increasing transmission (columns left to right) generally increases ROI, but only for strategies where testing starts young enough. Disparity (rows) combines the high-risk population size and how much additional exposure they endure. By the middle disparity setting, the high-risk individuals are effectively exposed every year, so shrinking that population in higher disparity settings must be balanced by increasing low-risk exposure probability to maintain the seropositivity rate; more low-risk exposure increases ROI opportunity. (Online version in colour.)

**Figure 3.**
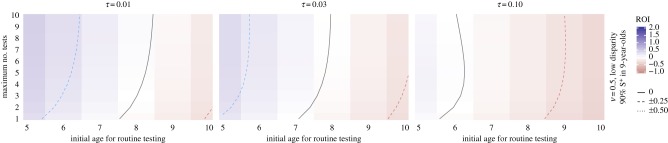
Sensitivity to number of tests. This area of epidemiological and economic parameters illustrates how there may be higher return with more tests (left-most), a minimum number required for positive return (middle) or a maximum (right-most), depending on test cost. (Online version in colour.)

We applied our model across a range of epidemiological settings and potential relative costs. Figure 2 shows test-then-vaccinate strategies that start after age 4, testing annually until a recipient is seropositive and thus vaccinated or reaches a maximum age (up to 20 in these results). For each epidemiological setting and relative cost, we report the lifetime value for the optimal strategy (i.e. the pair of initial and maximum testing ages that maximize ROI). We can view the same results from a different angle to understand trends in initial testing age and maximum number of tests. Figure 3 highlights the general trend that testing earlier is better, but this effect depends on epidemiological setting and costs. Some regions have a minimum number of tests for positive ROI; others, a maximum. We found trend reversions in several cases; while atypical, they highlight the need for context-specific analyses.

### 3.2. Model implementation and practical demonstration

While the general results suggest trends, they also identify the need for region-specific analyses. To that end, we implemented the model in an R package, denvax, which can be used with local epidemiological and economic data.

We demonstrate applying the approach with two practical examples for Malaysia and Peru, using epidemiological data from CYD14 [20] and a long-term study of Iquitos, Peru [21], and crude economic assumptions from previous studies [13]. We use the serological data to fit the two-risk, constant-FOI model, and then estimate the coefficients for equation (2.4). For this example, we assume disease and intervention costs, but decision-makers would estimate these from their regional data. Given local policy, disease cost might be based on a variety of measures, such as hospital costs, lost productivity or willingness to pay. Vaccination cost estimates could be based on quoted prices, and delivery costs derived from existing programmes with similar delivery strategies, e.g. human papillomavirus vaccine. Though costs are not yet available for an improved RDT, decision-makers could use the model to understand the maximum affordable test cost given other assumptions.

Here we assume that vaccination (i.e. full dose regimen and service delivery) costs US$78 per fully vaccinated child in both locations [13]. We assume testing costs of US$5 per recipient per year of testing (likewise full testing and service delivery). Finally, we assume the regions differ by secondary infection cost, using the South East Asian and Latin American societal costs from [13], US$86 and US$223, respectively. Thus for equation (2.4), the test and vaccine cost fractions are {*τ*, *ν*} = {0.06, 0.91} for Malaysia and {0.02, 0.35} for Peru.

**Figure 4.**
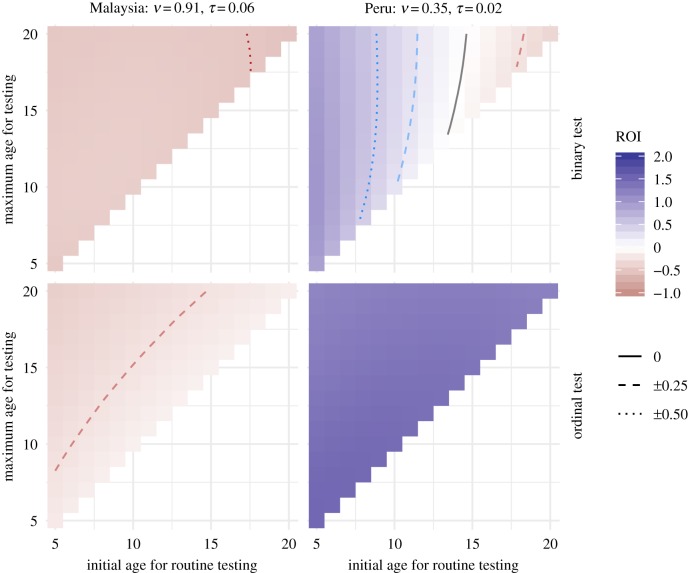
Practical comparison. ROI trends for two settings with seroprevalence between 70% and 80%. Using the assumed vaccination and testing costs, we find that a low secondary infection cost, *S*, and high exposure disparity (as assumed for Malaysia) results in negative ROI. However, in settings with high *S* and low exposure disparity (as assumed for Peru), ROI is positive when vaccination starts young enough. We show results for both binary and ordinal tests. While the more optimistic ordinal test can be substantially better (as shown for Peru-like results), that advantage may not be enough to make the intervention worthwhile (as shown in the Malaysia-like results). (Online version in colour.)

Since we are assuming costs, we focus on ROI for varying initial and maximum testing ages in figure 4. The settings produce very different surfaces, and highlight the importance of initial testing age when evaluating strategies. Given the demonstration data, no strategy produced a positive ROI in Malaysia, even with the ordinal test, and more testing only decreases ROI. Peru presents a different story: test-then-vaccinate strategies are beneficial if started young enough, and repeat testing improves ROI for all ages in the range considered, even for initial ages where single testing has a negative ROI.

## 4. Discussion

We demonstrated an approach that identifies CYD-TDV vaccination scenarios worthy of further investigation, and implemented a framework for local authorities to assess their potential return-on-investment using region-specific epidemiology and costs. It is impossible to provide universal answers to how or whether CYD-TDV should be used, but it is possible to describe a limiting relationship among a small set of factors and ascertain general trends.

We identified two such trends that should inform work on test-then-vaccinate strategies. First, repeat testing may improve cost-effectiveness over single testing; for some settings, this occurs even with relatively high testing costs. Second, we found routine test-then-vaccinate programmes often provide better ROI when targeting younger recipients, including those below the currently recommended 9 years old.

Since our model uses several optimistic assumptions, it is only appropriate for ruling out CYD-TDV in unfavourable settings, not for conclusively supporting its use. Justifying CYD-TDV requires more model realism. Future work should refine vaccine performance, which we know to be imperfect even in seropositive recipients and which has uncertain durability. Likewise, even the gold-standard tests for detecting prior dengue infection provide imperfect classification, particularly in the presence of other circulating arboviruses. Should a point-of-care RDT be licensed, it is unlikely to have better performance than the current gold standard [22]. Including these details will thus necessarily lead to lower ROIs than those estimated by our model.

However, incorporating insights from our model may help recover benefits. For example, we found annually repeating testing generally has superior performance, and under our assumptions biennial testing would be even better. This suggests repeat testing with lower frequency may be more cost-effective in more realistic models as well. Similarly, the substantial ROI improvement for younger intervention ages suggests that the current age guidelines for vaccination should be revisited if safety can be guaranteed by a highly specific test. Despite current licensing, CYD-TDV trials included younger participants and, after controlling for seropositivity, found safe and efficacious outcomes [15]; both the original and re-issued WHO guidance indicate that the 9-year-old limit is to ensure sufficiently high seroprevalence in the target population.

Accounting for these potential advantages properly will require more detailed data. Repeat testing may enable economies of scale (e.g. cheaper per unit tests), but will impose additional costs (e.g. record-keeping and service logistics). A model with repeat tests will also need to represent how individual test results are correlated. A lower routine intervention age will also affect test performance and vaccine efficacy. These concerns should be addressed in test development, so that data are available to inform future modelling work.

Multiple dengue vaccine candidates are currently in trials [23,24]. While they may prove durably tetravalent, given the complex dengue immunology and vaccine development history, there is a risk that these new vaccines will also have quirks. CYD-TDV, the only currently licensed vaccine, is flawed but reasonably understood and available now. We provide a clear tool to determine where it is not useful, but we also show that following the WHO-recommended test-then-vaccinate strategy for CYD-TDV can improve cost-effectiveness in many settings while satisfying clinical safety and ethical requirements, if a low-cost, suitable performance test can be developed. Whether that improvement is sufficient to warrant deploying the intervention will require additional work and detailed local analyses.

## Supplementary Material

Supplement

## Data accessibility

All relevant source code is available from https://gitlab.com/cabp_LSHTM/denvax/.
